# Autophagy is essential for maintaining the growth of a human (mini-)organ: Evidence from scalp hair follicle organ culture

**DOI:** 10.1371/journal.pbio.2002864

**Published:** 2018-03-28

**Authors:** Chiara Parodi, Jonathan A. Hardman, Giulia Allavena, Roberto Marotta, Tiziano Catelani, Marta Bertolini, Ralf Paus, Benedetto Grimaldi

**Affiliations:** 1 Department of Drug Discovery and Development, Laboratory of Molecular Medicine, Fondazione Istituto Italiano di Tecnologia (IIT), Genoa, Italy; 2 The Centre for Dermatology Research, University of Manchester, MAHSC, and National Institutes of Health Biomedical Research Center, Manchester, United Kingdom; 3 Monasterium Laboratory, Münster, Germany; 4 Department of Dermatology, University of Münster, Münster, Germany; 5 Department of Dermatology and Cutaneous Medicine, University of Miami Miller School of Medicine, Miami, Florida, United States of America; University of Oslo, Norway

## Abstract

Autophagy plays a crucial role in health and disease, regulating central cellular processes such as adaptive stress responses, differentiation, tissue development, and homeostasis. However, the role of autophagy in human physiology is poorly understood, highlighting a need for a model human organ system to assess the efficacy and safety of strategies to therapeutically modulate autophagy. As a complete, cyclically remodelled (mini-)organ, the organ culture of human scalp hair follicles (HFs), which, after massive growth (anagen), spontaneously enter into an apoptosis-driven organ involution (catagen) process, may provide such a model. Here, we reveal that in anagen, hair matrix keratinocytes (MKs) of organ-cultured HFs exhibit an active autophagic flux, as documented by evaluation of endogenous lipidated Light Chain 3B (LC3B) and sequestosome 1 (SQSTM1/p62) proteins and the ultrastructural visualization of autophagosomes at all stages of the autophagy process. This autophagic flux is altered during catagen, and genetic inhibition of autophagy promotes catagen development. Conversely, an anti–hair loss product markedly enhances intrafollicular autophagy, leading to anagen prolongation. Collectively, our data reveal a novel role of autophagy in human hair growth. Moreover, we show that organ-cultured scalp HFs are an excellent preclinical research model for exploring the role of autophagy in human tissue physiology and for evaluating the efficacy and tissue toxicity of candidate autophagy-modulatory agents in a living human (mini-)organ.

## Author summary

Human scalp hair follicles (HFs) experience a massive growth for years, until they spontaneously enter into a rapid, apoptosis-driven organ involution process, orchestrated by an organ-intrinsic “hair cycle clock,” the molecular control of which remains unclear. Human HFs maintain in vivo–like characteristics, even after being removed from the body, and spontaneously run through a fundamental organ-remodelling process, traversing through a stage of growth (anagen) and destruction (catagen) as a (mini-)organ model. Here, we exploit this unique remodelling (mini-)organ to unveil a crucial new role of autophagy in the growth of human HFs. We show that hair matrix keratinocytes exhibit an active autophagic flux ex vivo during anagen, which is altered after the transition to catagen. We find that genetic inhibition of follicular autophagy induces premature catagen and enhances hair matrix keratinocyte apoptosis, suggesting that autophagic flux in the anagen hair matrix is important for the maintenance of this stage. Indeed, we find that the principal ingredients of a product used to treat hair loss induces autophagy in organ-cultured human scalp HFs and promotes anagen. We conclude that organ-cultured human HFs are a suitable (mini-)organ system to study both the role of autophagy in human physiology ex vivo and to test candidate agents that modulate autophagy under clinically relevant conditions.

## Introduction

In recent years, autophagy has emerged as a pivotal actor in adaptive responses to stress and starvation [1–3] and in tissue homeostasis [4], cellular differentiation [5], and ageing [6,7]. Key concepts of autophagy have arisen from molecular genetic experiments in a number of model organisms, including mammals, in vivo and ex vivo [8].

However, the role of autophagy in human organ physiology is as yet incompletely understood due to the lack of human model systems and the difficulty of experimental manipulation. For this, it would be helpful to have an easily tractable, clinically relevant human organ model at our disposal. Moreover, such human models would be useful to assess the efficacy and safety of the ever-increasing number of strategies that are being proposed to therapeutically modulate autophagy to treat various human diseases and to slow tissue ageing [9–12].

On this background, we have turned to a complete, cyclically remodelled human (mini-)organ, i.e., terminal scalp hair follicles (HFs) [13]. Human HFs can be easily microdissected from excess tissue removed during plastic or hair transplantation surgery and organ cultured in a well-defined, supplemented, serum-free medium [14]. Under these conditions, growing (anagen) HFs continue to produce a pigmented hair shaft and will continue their spontaneous organ remodelling activity for many days ex vivo.

The organ culture of human HFs has not only permitted major advances in translational hair research but have also permitted novel insights into human tissue physiology and pathology, spanning diverse fields including metabolism, cellular differentiation, chronobiology, cell cycle control, immunology, (neuro)endocrinology, toxicology, and pharmacology [15]. Therefore, the value of HF organ culture as a model for biomedical research extends far beyond its importance for dermatology alone.

After years of massive growth activity (anagen), human scalp HFs spontaneously enter into a rapid, apoptosis-driven organ involution process (catagen) [16], following the dictates of an as yet insufficiently understood, organ-intrinsic “hair cycle clock” [17–19]. We hypothesized that late-stage anagen scalp HFs, whose hair matrix epithelium proliferates at a higher rate than most malignant tumors, despite being exposed to a number of stressors, are likely to come under increasing pressure to maintain tissue homeostasis and may require a substantial autophagic flux [20] to maintain their growth.

That the HF can recover from massive toxicological insults, such as during chemotherapy-induced alopecia [21], and that the antimalarial agent chloroquine (CQ), a major autophagy inhibitor used in the clinic, can elicit adverse hair effects, such as change in hair color and hair loss [22], also encouraged the concept that the HF may engage in autophagy as a fundamental adaptive mechanism against stress.

Here, we have tested the hypotheses that organ-cultured human scalp HFs need to maintain a substantial autophagic flux in order to sustain anagen and that these (mini-)organs are well suited to study both the role of autophagy in human organ physiology ex vivo and to test candidate agents that modulate autophagy in a therapeutically desired manner under clinically relevant conditions. In the following, we report evidence that confirms both working hypotheses.

## Results

### Organ-cultured HF are a suitable assay system for studying autophagy in a human (mini-)organ ex vivo

The yeast homologous autophagy-related protein 8 (ATG8), Light Chain 3 (LC3), and sequestosome 1 (SQSTM1, also known as p62) are well-documented markers to monitor autophagy by fluorescence microscopy [23]. Lipid conjugated LC3 proteins (LC3-II) are specifically recruited on the membrane of autophagosomes from the initial stages of autophagy [24]. Differing from a diffuse cytoplasmic signal of the unconjugated LC3 form (LC3-I), lipidated LC3-II–containing autophagosomes appear as fluorescent dots when assessed by indirect immunofluorescence (IF) [23]. However, visualizing the endogenous LC3 protein in compact tissues can be quite challenging [23]. Therefore, as a first step in characterizing the role of autophagy in cycling human HFs, we first established a suitable indirect IF microscopy protocol to detect LC3 in acetone-fixed cryosections of organ-cultured anagen HFs. For this, we used a specific anti-LC3B antibody that has a higher affinity for the lipidated LC3B form (S1 Fig).

**Fig 1:**
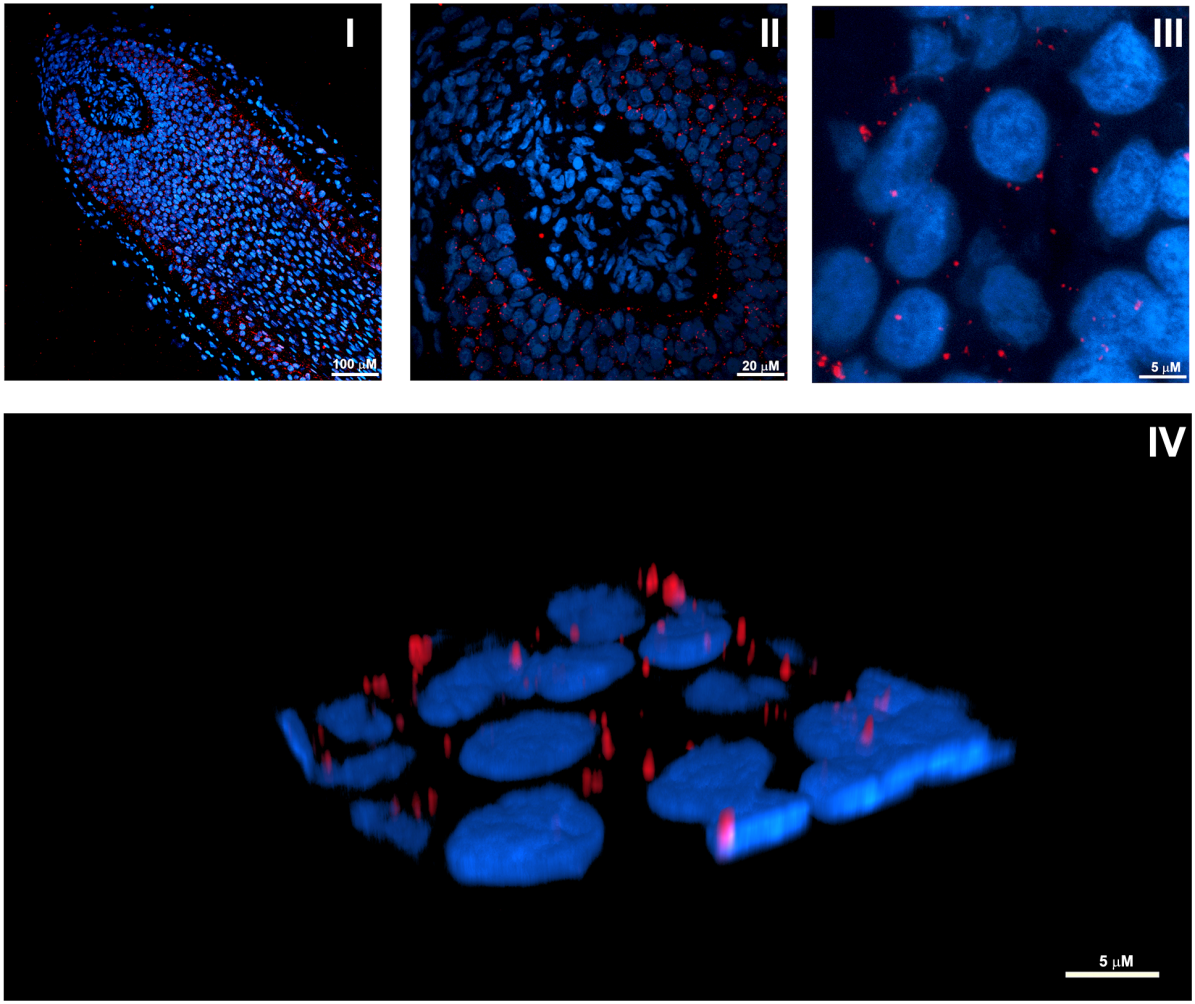
IF visualization of autophagy in anagen hair MKs from organ-cultured human HFs. (I) Representative confocal image of organ-cultured anagen HFs probed with a specific anti-LC3B antibody/Alexa555 (red) and Hoechst 33342 nuclear staining (blue). (II and III) High magnification images showing the predominance of LC3B-positive dots in keratinocytes of the most proximal hair matrix and the precortical hair matrix. (IV) Three-dimensional reconstruction from high resolution confocal images showing the perinuclear distribution of LC3B-positive fluorescent dots, consistent with the recognized cellular localization of autophagosomes [23]. HF, hair follicle; IF, immunofluorescence; LC3B, Light Chain 3B; MK, matrix keratinocyte.

Confocal microscopy with anti-LC3B antibody/Alexa555 (red) demonstrated the presence of cells with distinct perinuclear fluorescent signal (Fig 1III and 1IV), consistent with the recognized cellular localization of autophagosomes [23].

Interestingly, LC3B-positive dots were most prominently seen in keratinocytes of the proximal hair matrix below Auber’s line, the most rapidly proliferating compartment of the HF epithelium [25], and in the precortical hair matrix (Fig 1I and 1II), i.e., the epithelial compartment where undifferentiated HF keratinocytes become committed to undergo terminal differentiation into the cells of the inner root sheath, hair shaft cortex, or medulla and begin synthesizing large quantities of specific hair keratins [26].

**Fig 2:**
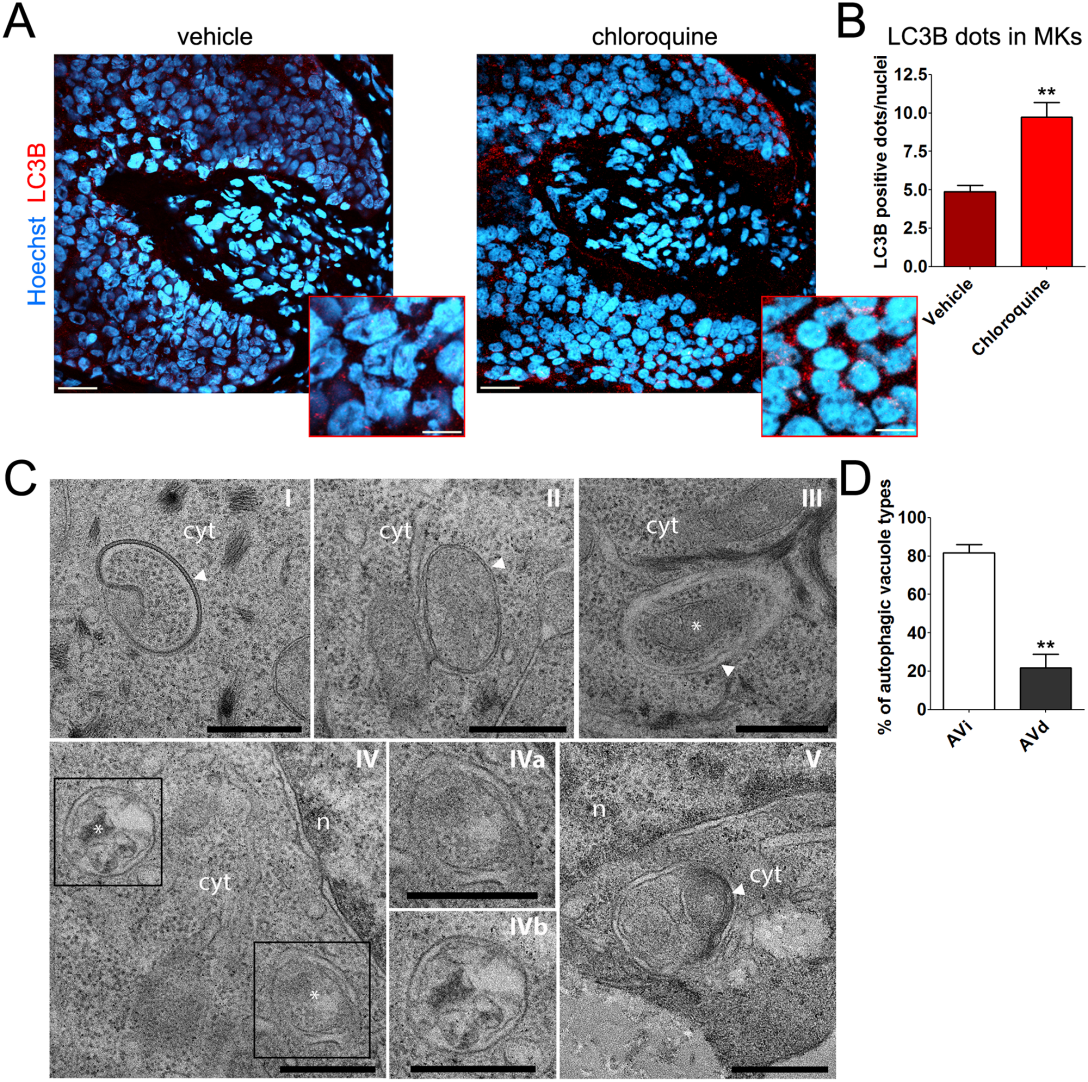
Intrafollicular autophagic flux is active in MKs. (A) Representative confocal images of organ-cultured anagen HFs treated for 4 h with CQ (10 μM) or vehicle (water). An overlay of LC3B/Alexa555 (red) and Hoechst 33342 (blue) is shown (scale bar 20 μm). The boxed regions show high magnification images of LC3B-fluorescent dots in MKs (scale bar 5 μm). (B) CQ treatment significantly increased the number of LC3B-flurescent dots per nuclei in the hair matrix region compared with vehicle. Quantification was only performed on epithelial HF cells, specifically the hair matrix cells and precortical matrix, while the connective tissue sheath and dermal papilla were excluded from analysis. Shown as average of LC3B-fluorescent dots/nuclei ± SEM. **P < 0.01, CQ versus vehicle. (C) Representative TEM images from organ-cultured anagen HFs showing a continuous autophagic flux in MKs. (I–III) AVi’s with typical, well-visible bilayers separated by a narrower electron-lucent cleft (arrowheads). Predominantly, these AVi’s contained morphologically intact cytosol and organelles. Note the well-defined mitochondria in III (asterisk). (IV) AVd’s characterized by a partially or completely degraded internal membrane and electron dense cytoplasmic material and/or organelles at various stages of degradation (asterisks). IVa and IVb are higher magnifications of the boxed regions in IV. (V) Putative autophagolysosome characterized by lamellar internal membranes (arrowhead). Scale bars are 0.5 μm in C-I, C-III, C-IV, C-Iva, and C-IVb; 1 μm in C-II and C-V. (D) Quantitative assessment of autophagic structures in TEM sections showing the relative percentage of AVi and AVd in anagen HFs. Shown as average ± SEM. **P < 0.01, AVd versus AVi. The underlying numerical data and statistical analysis for panels B and D are provided in S1 Data. AVd, late/degradative autophagic vacuole; AVi, initial autophagic vacuole; CQ, chloroquine; cyt, cytoplasm; HF, hair follicle; LC3B, Light Chain 3B; MK, matrix keratinocyte; n, nucleus; TEM transmission electron microscopy.

To further support that punctate fluorescent signals do indeed correspond to LC3B-containing autophagosomes, we conducted confocal microscopy analysis in anagen organ-cultured HFs that were treated for 4 h with CQ (10 μM) with a vehicle control. Because CQ blocks autophagy at its late stage by inhibiting lysosomal function, CQ induces the accumulation of autophagolysosomes enclosing lipidated LC3B [27]. Compared with vehicle-treated samples, confocal images of CQ-treated HFs showed a significant increase in the number of LC3B-positive fluorescent dots (red, Alexa555), primarily in matrix keratinocytes (MKs) (Fig 2A and 2B).

Thus, our IF method is suitable to monitor endogenous autophagy in human organ-cultured HFs by indirect fluorescence microscopy. Moreover, the fact that LC3B-fluorescent dots increased upon CQ treatment indicates that the intrafollicular autophagic flux was active in MKs and that autophagolysosomal function was preserved in cultured human HFs [23].

Next, these IF results were independently investigated by transmission electron microscopy (TEM). These ultrastructural analyses supported the presence of an active autophagic flux in the human HF matrix by showing diverse cytoplasmic double-membrane structures belonging to autophagic vacuoles at different stages of autophagosome biogenesis [28] (Fig 2C and 2D). Indeed, we observed a putative phagophore sequestering a portion of the cytoplasm to form an autophagosome (Fig 2CI). We also recognized several initial autophagic vacuoles (AVi’s) with visible bilayers separated by a narrower electron-lucent cleft, typical of autophagy (Fig 2CII, 2CIII and 2CIVa). Predominantly, these AVi contained morphologically intact cytosol with ribosomes and organelles (see as an example the mitochondria in Fig 2CIII), which is a common feature of nonselective autophagy [23]. In addition, we observed late/degradative autophagic vacuoles (AVd’s) characterized by a partially or completely degraded internal membrane and electron dense cytoplasmic material and/or organelles at various stages of degradation (Fig 2CIVb). We also recognized a putative autophagolysosome, i.e., degradative autophagic vacuole, that has fused with a lysosome, characterized by lamellar internal membranes (Fig 2CV). Quantification of TEM images showed no accumulation of AVd structures (Fig 2D), supporting the notion that the autophagic flux in the matrix of human anagen HFs ex vivo is actively occurring.

### Autophagic flux is altered during the anagen-to-catagen transformation of human HFs

**Fig 3:**
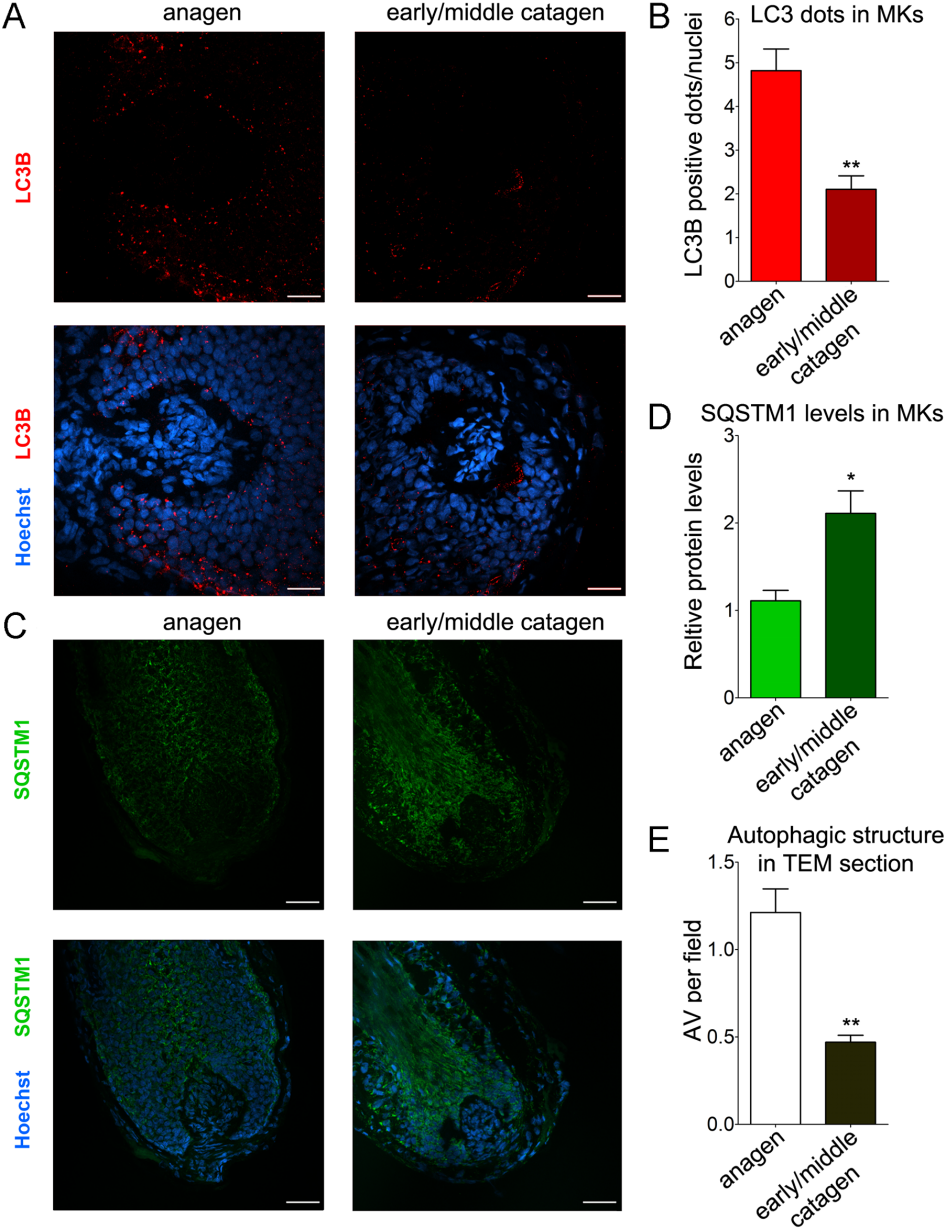
Autophagy is altered during the transition from anagen to catagen. (A and B) Confocal fluorescence analysis revealed a marked reduction in the number of LC3B-positive fluorescent dots during the transition from anagen to catagen. (A) Representative confocal images of anagen and early/middle catagen organ-cultured HFs probed with specific anti-LC3B antibody/Alexa555 (red). Nuclei were stained with Hoechst 33342 (blue). (B) Quantification of the number of LC3B-flurescent dots per nuclei in the hair matrix region of anagen and early/middle catagen HFs, shown as the average of LC3B-fluorescent dots/nuclei ± SEM. **P < 0.01, catagen versus anagen. (C and D) In line with a reduction in autophagy-dependent protein degradation during HF cycle progression into catagen, the protein levels of SQSTM1 significantly increases in catagen. (C) Representative confocal images of anagen and early/middle catagen organ-cultured HFs probed with specific anti-SQSTM1 antibody/Alexa488 (green). Nuclei were stained with Hoechst 33342 (blue). (D) Quantification of SQSTM1-fluorescent signal in the hair matrix region of anagen and catagen HFs. Fluorescent intensity of anagen HFs was set to 1. Shown as relative integrated density ± SEM. *P < 0.05, catagen versus anagen. (E) Quantitative assessment of autophagic structures in TEM sections from anagen and catagen HFs showing the relative percentage of AVs per field. Shown as average ± SEM. **P < 0.01, catagen versus anagen. All quantifications were performed only on epithelial HF cells, specifically the hair matrix cells and precortical matrix, while the connective tissue sheath and dermal papilla were excluded from analysis. The underlying numerical data and statistical analysis for panels B, D, and E are provided in S1 Data. AV, autophagic vacuole; HF, hair follicle; LC3B, Light Chain 3B; MK, matrix keratinocyte; SQSTM1, sequestosome 1; TEM, transmission electron microscopy.

Next, we asked whether key autophagy readout parameters change when human anagen scalp HFs spontaneously enter into catagen ex vivo [15] by comparing anagen VI HFs with HFs that showed morphological criteria of early or middle catagen stages, using a battery of previously defined objective classification criteria [29]. Confocal images revealed a marked reduction in the number of LC3B-positive fluorescent dots during the transition from anagen to catagen (Fig 3A and 3B), suggesting a more prominent autophagic flux during the proliferative stage of the HF cycle.

To probe this hypothesis, we compared the levels of SQSTM1 in anagen and catagen HFs. SQSTM1 serves as a link between LC3 and ubiquitinated substrates [30]. SQSTM1 and SQSTM1-bound polyubiquitinated proteins become incorporated into the completed autophagosome and are degraded in autolysosomes, thus serving as an index of autophagic degradation [23]. In line with a reduction in autophagy-dependent protein degradation during catagen induction, confocal images showed that SQSTM1 fluorescence signal (green, Alexa488) was significantly higher in the matrix of catagen HFs, compared with the matrix of HFs in anagen VI (Fig 3C and 3D).

Further supporting our IF data, quantitative assessment of autophagy-related structures in TEM sections from anagen and catagen HFs showed a significantly higher number of autophagic vacuoles in anagen versus catagen HFs (Fig 3E).

### Autophagy inhibition promotes catagen development

These observations raised the possibility that autophagy may serve as a process that maintains and prolongs anagen. If true, manipulating intrafollicular autophagy would be of profound clinical interest, because the vast majority of patients with hair loss or undesired hair growth seen in clinical practice shows a premature shortening of anagen (leading to effluvium/alopecia) or retarded entry into catagen (resulting in hirsutism/hypertrichosis) [13,19].

As mentioned above, treatments with the autophagy inhibitor CQ present recognized deleterious effects on hair viability [22]. Nevertheless, CQ may eventually affect the hair cycle through autophagy-independent cytotoxic effects related to the ability of this drug to inhibit lysosomal function or to intercalate in DNA [31,32]. Therefore, to evaluate whether autophagy has a pro-anagen function, we adopted a molecular genetic approach by knocking down the autophagy-related gene 5 (*ATG5*) with iRNA, a gene that plays a fundamental role in the early stages of autophagosome formation [33].

**Fig 4:**
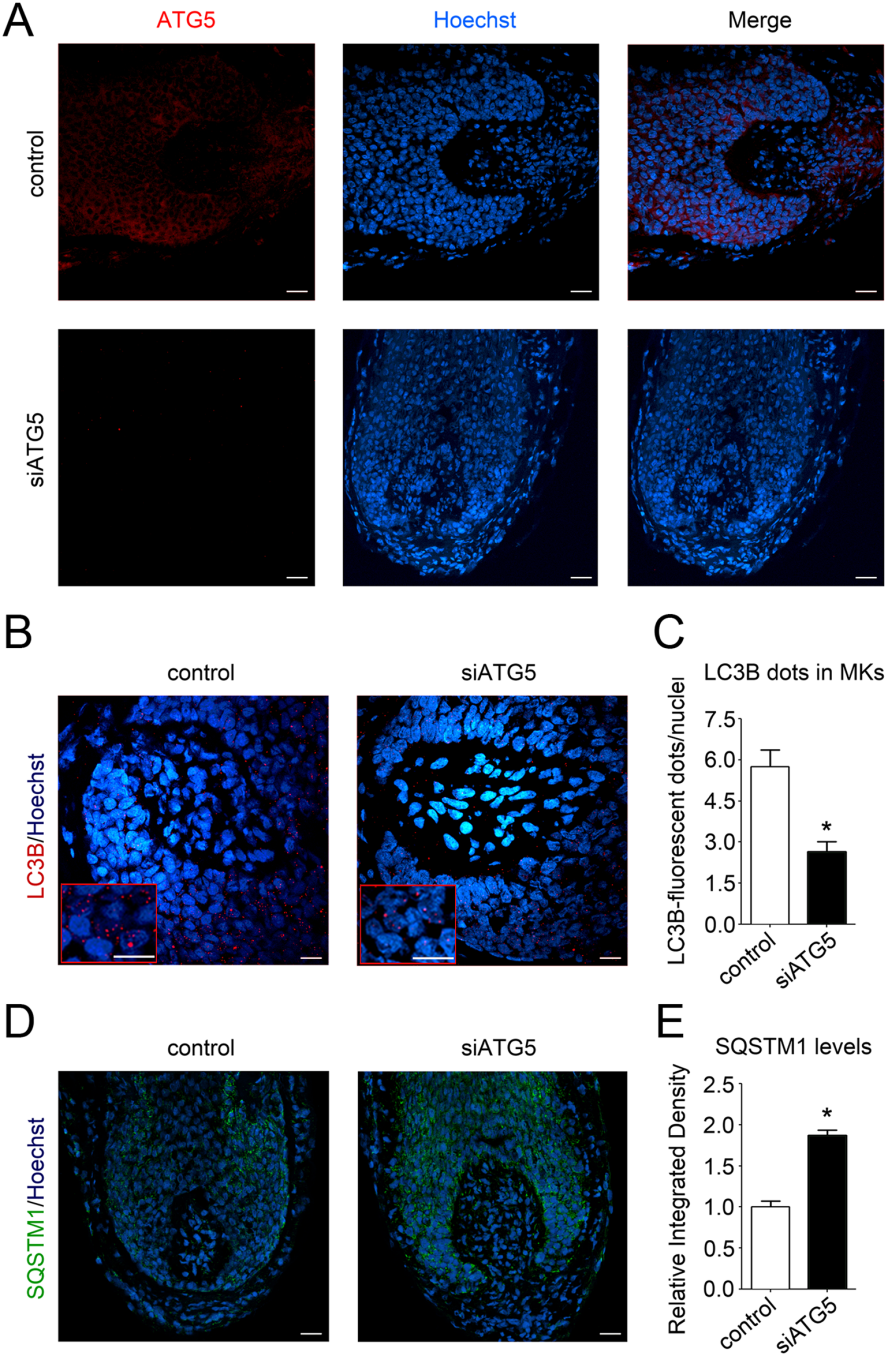
Silencing of the essential autophagy gene, *ATG5*, functionally reduces intrafollicular autophagy. (A) Organ-cultured HFs were transfected with pooled siRNA sequences against the autophagy gene, *ATG5* (siATG5), or a nontargeting pool (Control). Forty-eight hours after transfection, HFs were processed for indirect IF analysis with a specific anti-ATG5 antibody/Alexa555 (red). Hoechst 33342 was used to stain nuclei (blue). Demonstrating the efficient knock-down of ATG5 in HFs, ATG5-fluorescence signals were undetectable in siATG5 HFs. (B–E) ATG5 silencing actually impaired autophagy in organ-cultured HFs. (B) Representative confocal images of siATG5 and control HFs probed with specific anti-LC3B antibody/Alexa555 (red). Nuclei were stained with Hoechst 33342 (blue). Insets show high magnification of LC3B-fluorescent dots in MKs. Scale bars are 10 μm. (C) Quantification of the number of LC3B-fluorescent dots per nuclei in the hair matrix region of siATG5 and control HFs. Shown as average of LC3B-fluorescent dots/nuclei ± SEM. *P < 0.05, siATG5 versus control HFs. (D) Representative confocal images of siATG5 and control organ-cultured HFs probed with specific anti-SQSTM1 antibody/Alexa488 (green). Nuclei were stained with Hoechst 33342 (blue). Scale bars are 20 μm. (E) Quantification of SQSTM1-fluorescent signal in hair matrix region of siATG5 and control HFs. Fluorescent intensity of control HFs was set to 1. Shown as relative integrated density ± SEM. *P < 0.05, siATG5 versus control HFs. All quantifications were performed only on epithelial HF cells, specifically the hair matrix cells and precortical matrix, while the connective tissue sheath and dermal papilla were excluded from analysis. The underlying numerical data and statistical analysis for panels C and E are provided in S1 Data. *ATG5*, autophagy-related gene 5; HF, hair follicle; IF, immunofluorescence; MK, matrix keratinocyte; siRNA, small interfering RNA; SQSTM1, sequestosome 1.

Anagen HFs from three diverse human individuals were transfected with pool small interfering RNA (siRNA) sequences against *ATG5* (siATG5) or with nontargeting scrambled oligonucleotides, using our previously described transfection method for gene silencing in human HFs [34,35]. Confirming the silencing was successful, 48 h after transfection, the ATG5-fluorescent signal was drastically reduced in siATG5-treated HFs compared with control HFs (Fig 4A).

Additionally, the number of LC3B-positive dots was significantly reduced in siATG5 HFs, demonstrating that ATG5 silencing was functionally deleterious to intrafollicular autophagy (Fig 4B and 4C). Further confirming a decrease in autophagic degradation when ATG5 was silenced, SQSTM1 fluorescence levels were elevated in siATG5 HFs, compared with control HFs (Fig 4D and 4E).

**Fig 5:**
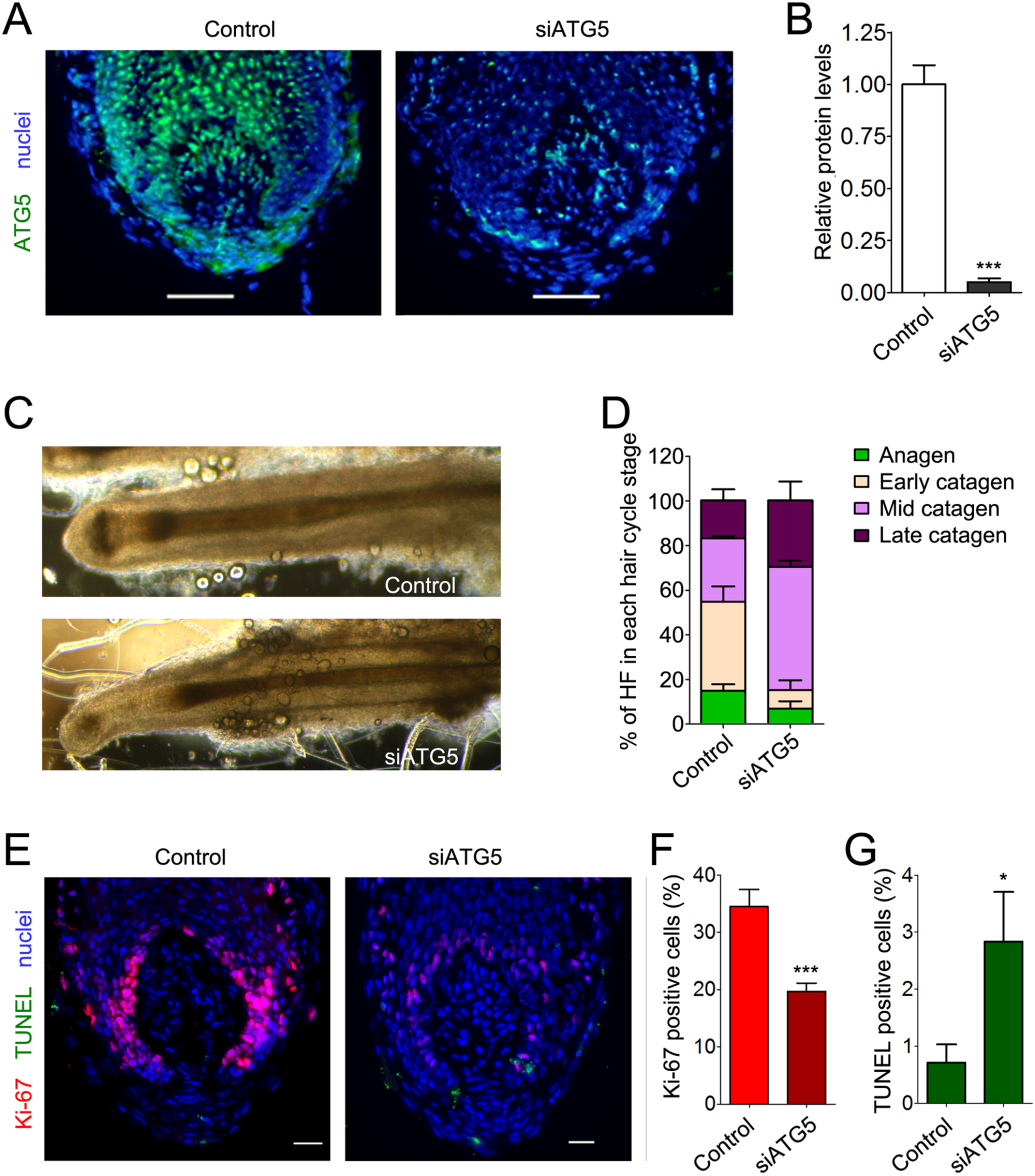
Genetic inhibition of autophagy in organ-cultured anagen HFs prematurely induces catagen and promotes hair MK apoptosis. (A and B) Efficiency of ATG5 silencing persists at 96 h post-transfection, as evaluated by indirect IF. (A) Representative fluorescence image of siATG5 and control HFs probed with a specific anti-ATG5 antibody/Alexa488 (green). Hoechst 33342 was used to stain nuclei (blue). Shown as overlay between ATG5- and DAPI-positive signals. (B) Quantification of ATG5-fluorescent signals in siControl (Control) and siATG5 HFs. ***P < 0.001, siATG5 versus Control. (C and D) The hair cycle stage of numerous HFs from independent individuals at 96 h post-transfection with siRNA against ATG5 or scramble (Control) was assessed by morphological analysis using a battery of previously defined objective classification criteria [29]. (C) A representative image showing the morphology of control and siATG5 HFs. (D). The percentage of HFs at the different stages of the hair cycle was evaluated and reported as percentage of HFs in each hair cycle stage ± SEM. (E–G) Evaluation of the number of proliferative and apoptotic MKs below the widest part of the dermal papilla (Auber’s line) in control and siATG5 HFs. Proliferative cells were assessed by immune reactivity reactive toward the proliferative marker, Ki-67 (red, Alexa555), while apoptotic cells were determined by TUNEL (green). Hoechst 33342 was used to stain nuclei (blue). A representative image showing the overlay of Ki-67–, TUNEL-, and Hoechst-positive signals in control and ATG5-silenced HFs is provided in (E). (F and G) The percentage of Ki-67– and TUNEL-positive cells was calculated and reported as mean ± SEM. ***P < 0.001 and *P < 0.05, siATG5 versus Control. As TUNEL-positive cells in the dermal papilla and connective tissue sheath are a well-recognized artifact of HF organ culture [15,16,29], intramesenchymal TUNEL-positive cells were excluded from the quantitative analysis. The underlying numerical data and statistical analysis for panels B, D, F, and G are provided in S1 Data. ATG5, autophagy-related gene 5; HF, hair follicle; IF, immunofluorescence; MK, matrix keratinocyte; siRNA, small interfering RNA; TUNEL, Terminal deoxynucleotidyl transferase dUTP Nick End Labeling.

We then morphologically and immunohistologically assessed the hair cycle stage of each HF 96 h after transfection with siRNA sequences against *ATG5* or Control, as previously described [29]. Confirming that ATG5 silencing persisted at this time point, IF analysis showed a significant 80% reduction in ATG5-fluorescence signal of siATG5-treated HFs, compared with control HFs (Fig 5A and 5B).

While the majority of control HFs progressed through the anagen-catagen transformation relatively slowly and were mostly in early catagen stage, siATG5-transfected HFs involuted much more rapidly and reached the middle and late stages of catagen development (Fig 5C and 5D) with less than 10% of ATG5-silenced HFs having retained their characteristic anagen VI morphology.

To validate these morphological analyses, we assessed the hair cycle stage in siATG5 and control HFs by measuring the number of proliferative and apoptotic MKs in the hair matrix tips, below the line that represents the widest part of the hair bulb (Auber’s line) [15,29]. Compared with control HFs, we observed a significant reduction in the percentage of MKs that were positive for the proliferation marker, Ki-67 (red, Alexa555), in siATG5-treated HFs, with a significantly higher percentage of apoptotic (Terminal deoxynucleotidyl transferase dUTP Nick End Labeling [TUNEL]-positive) cells (green, Alexa488) (Fig 5E–5G). Therefore, experimental autophagy inhibition prematurely terminates anagen and promotes apoptosis-driven development.

### A commercial product used to treat hair loss acts as a potent inducer of intrafollicular autophagy

The above findings imply that, conversely, up-regulating intrafollicular autophagy should prolong anagen. Notably, the principal ingredients (core mix) of an anti–hair loss product on the market contains *Galeopsis segetum* extract, biotin, and N^1^-methyspermidine, the latter of which is a metabolically stable analog of the well-recognized autophagy-promoting agent, spermidine [36–39].

**Fig 6:**
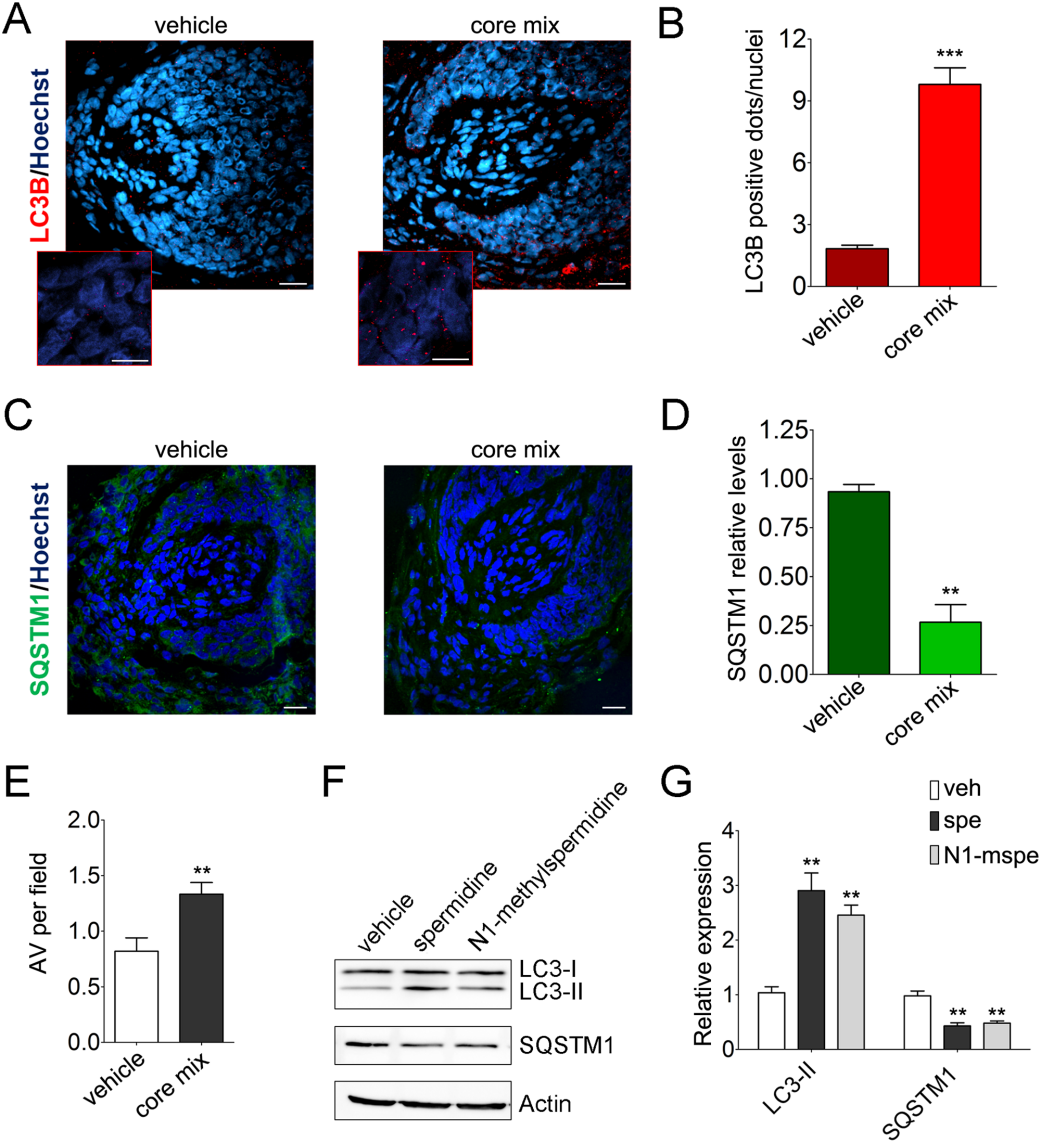
The principal ingredients of an anti–hair loss product act as a potent inducer of intrafollicular autophagy. (A) Representative confocal images of anagen organ-cultured HFs treated with the principal ingredients (core mix) of the topic formulation of an anti–hair loss product on the market, which includes N^1^-methyspermidine, a metabolically stable analog of the well-recognized autophagy-promoting agent, spermidine [36–39]. An overlay of LC3B/Alexa555 (red) and Hoechst 33342 (blue) is shown. Scale bars are 20 μm. Insets show high magnification of LC3B-fluorescent dots in MKs (scale bar 5 μm). (B) Quantification of the number of LC3B-fluorescent dots per nuclei in the hair matrix region of core mix–and vehicle-treated HFs (connective tissue sheath and dermal papilla were excluded from analysis), shown as average of LC3B-fluorescent dots/nuclei ± SEM. ***P < 0.001, core mix versus vehicle. (C) Representative confocal images of core mix–and vehicle-treated HFs probed with specific anti-SQSTM1 antibody/Alexa488 (green). Nuclei were stained with Hoechst 33342 (blue). Scale bars are 20 μm. (D) Quantification of SQSTM1-fluorescent signal in the hair matrix region of HFs treated with core mix or vehicle. Fluorescent intensity of vehicle HFs was set to 1. Shown as relative integrated density ± SEM. **P < 0.001, core mix versus vehicle. (E) Quantitative assessment of autophagic structures in TEM sections from HFs treated with core mix or vehicle. Shown as average number of AVs per field ± SEM. *P < 0.05, core mix versus vehicle. (F and G). Pro-autophagic activity of N^1^-methyspermidine was tested in an in vitro cellular assay previously adopted to demonstrate the ability of spermidine in inducing autophagy [36]. Cultured human U2OS cells were treated 6 h with vehicle or equimolar doses of spermidine and N^1^-methyspermidine (100 μM). (F) The levels of lipidated LC3B (LC3-II) and SQSTM1 were then assessed by immunoblotting analysis with specific antibody. Actin signals were adopted as a loading control. (G) Densitometry analysis of protein signals is reported as relative protein levels normalized by ACTIN. Vehicle sample value was set to 1. Shown as mean ± SEM, *n* = 3; **P < 0.01, compounds versus vehicle. The underlying numerical data and statistical analysis for panels B, D, E, and G are provided in S1 Data. ACTIN, actin beta; AV, autophagic vacuole; HF, hair follicle; LC3, Light Chain 3; LC3B, Light Chain 3B; MK, matrix keratinocyte; spe, spermidine; SQSTM1, sequestosome 1; TEM, transmission electron microscopy; U2OS, human bone osteosarcoma epithelial U2OS cell line; veh, vehicle.

We thus decided to test whether this core mix was able to induce autophagy and prolong anagen in organ-cultured HFs. Confocal images showed a marked increase of LC3B-positive fluorescent dots in HFs treated with the core mix, compared with vehicle-treated HFs (Fig 6A and 6B).

Supporting this, the core mix treatment significantly lowered SQSTM1-fluorescent signal compared with the vehicle, demonstrating that the increase in LC3B-fluorescent autophagosomes depended on an increased autophagic flux, thus enhanced autophagy-mediated degradation (Fig 6C and 6D). Quantification of autophagic structures in TEM sections from HFs treated with core mix or a vehicle further validated an induction of a bona fide autophagic flux upon treatment (Fig 6E).

This result also suggests that the N^1^-methylspermidine retains the ability to induce autophagy, as its desmethylated analog. To validate this concept, we adopted an in vitro cellular assay to demonstrate the pro-autophagic function of spermidine [36]. We thus evaluated the levels of lipidated LC3B and SQSTM1 in human bone osteosarcoma epithelial U2OS cells treated with equimolar doses of N^1^-methyspermidine and spermidine. Consistent with the results published by Pietracola and coworkers [36], spermidine treatment increased the levels of the lipidated LC3B-II form and stimulated autophagy-mediated degradation of SQSTM1 (Fig 6F and 6G). In addition, N^1^-methylspermidine–related effects on both LC3B-II and SQSTM1 levels paralleled the differences observed with the natural spermidine version (Fig 6F and 6G). Further indicating that both compounds can also induce autophagy in keratinocytes, these results were extended and confirmed in the human NCTC 2544 keratinocyte cell line (S2 Fig).

**Fig 7:**
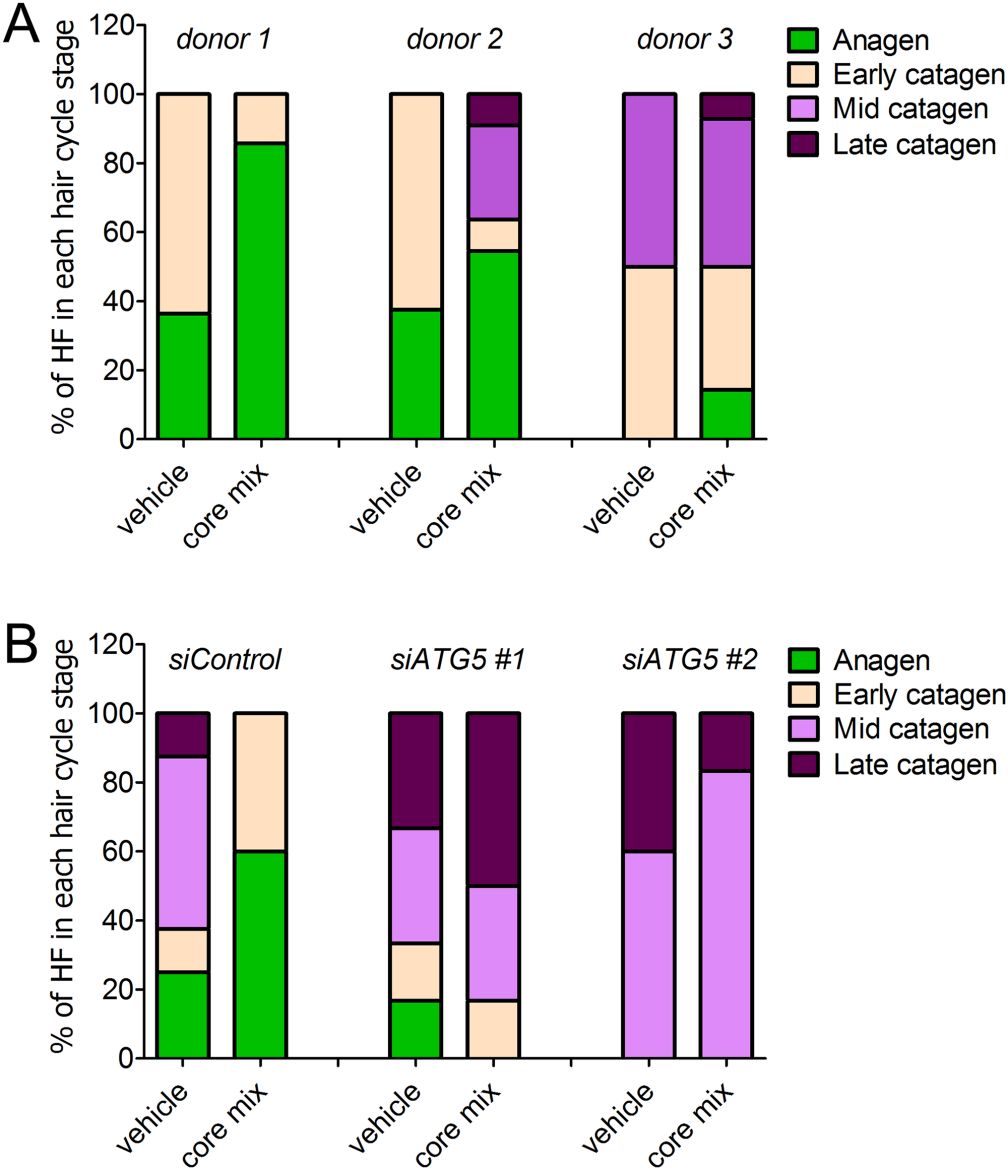
Core mix–related autophagic stimulation is instrumental in its anagen promoting activity. (A) Pro-anagen activity of the principal ingredients of an anti–hair loss product on the market (core mix). The hair cycle stage of anagen VI HFs from diverse donors treated with core mix or vehicle was assessed by morphological analysis [29]. Compared with vehicle, core mix treatment increased the percentage of anagen HFs (green) from all donors. Notably, the anagen-promoting effects of the core mix was observed even in catagen-primed HFs (donor 3), as indicated by the fact that all vehicle-treated HFs had transitioned into catagen at the end of organ culture. (B) Pro-anagen activity of the core mix is impaired in HFs silenced for the essential autophagy gene, *ATG5*. Anagen VI HFs from three donors were transfected with pool siRNA sequences against *ATG5* gene (*siATG5 #1 and ATG5 #2*) or with nontargeting scrambled oligonucleotides (*siControl*) and then treated with core mix or vehicle. Consistent with the analysis shown in (A), subsequent evaluation of the hair cycle stage revealed that siControl HFs treated with the core mix had an increased percentage of anagen HFs compared with vehicle-treated HFs. In marked contrast, core mix treatment failed to promote anagen in ATG5-silenced HFs from two independent donors. The underlying numerical data and statistical analysis are provided in S1 Data. *ATG5*, autophagy-related gene 5; HF, hair follicle; siRNA, small interfering RNA.

Next, we decided to validate that the autophagy-enhancing ability of the core mix corresponded to a pro-anagen effect. We thus assessed the hair cycle stage of anagen HFs from three diverse human donors after a 5-d treatment with the core mix or vehicle. Despite a substantial variability in HF cycling among the diverse donors frequently observed in human HF organ culture experiments in which multiple patients were used [15], morphological evaluation showed that the treatment with the core mix increased the percentage of anagen HFs from all donors (Fig 7A).

Moreover, the autophagy-inducing mix significantly enhanced the relative percentage of Ki-67 proliferative cells while reducing apoptotic MKs in the hair matrix tips, below the Auber’s line (S3 Fig). Notably, the anagen-promoting effects of the core mix was observed even in catagen-primed HFs. Indeed, the treatment of anagen VI HFs from a donor that was already primed to enter catagen, as indicated by the fact that all vehicle-treated HFs had transitioned into catagen at the end of organ culture, preserved a clear anagen VI morphology in 16% of core mix–treated HFs (Fig 7A, donor 3).

To further validate that the observed promotion of anagen was related to an induction of autophagy, we repeated the treatment in HFs silenced for the ATG5 gene. Supporting our previous analysis, siControl HFs treated with the core mix had an increased percentage of anagen HFs compared with vehicle-treated HFs (Fig 7B, *siControl*). In marked contrast, core mix treatment failed to promote anagen in ATG5-silenced HFs from two independent donors (Fig 7B, *siATG5 #1* and *siATG5 #2*).

Collectively, our results support a scenario in which intrafollicular autophagy plays a fundamental anagen-maintaining role in HFs.

## Discussion

The current study unveils a crucial new role of autophagy in human hair growth control, namely for maintaining the HF growing stage, anagen. Moreover, we show that organ-cultured scalp HFs are an excellent preclinical research model for exploring autophagy functions in human tissue physiology and for evaluating the efficacy and tissue toxicity of candidate autophagy-promoting and -inhibitory agents in a living human (mini-)organ.

Specifically, we present the first evidence that anagen hair MKs exhibit an active autophagic flux ex vivo, as documented by the presence of several LC3B-fluorescent perinuclear dots (Fig 1), which increase upon CQ treatment (Fig 2A and 2B), and by demonstrating that autophagosomes representing different stages of autophagy are present in hair MKs (Fig 2C and 2D).

We further show that the number of autophagosomes decreases during the spontaneous involution of this (mini-)organ (catagen) (Fig 3), suggesting that intrafollicular autophagy may be modulated by several factors that are also involved in the regulation of HF cycling [40]. Because fibroblast growth factor (FGF) signaling is an important positive regulator of autophagy in chondrocytes [41] and is also involved in hair cycle control [40], FGFs are among the most plausible regulators of intrafollicular autophagy. For example, fibroblast growth factor FGF7 (also known as keratinocyte growth factor [KGF]) is predominantly expressed in anagen and protects human HF from cell death induced by UV irradiation and chemotherapeutic or cytotoxic agents [42], while FGF5 signaling controls catagen development [40]. Such candidate regulators of HF autophagy can now be probed in HF organ culture, using the methods and readouts reported here.

Interestingly, the molecular controls that govern the anagen-catagen transformation in human HFs include profound changes in intrafollicular peripheral clock activity, whereby clock silencing prolongs anagen [18]. Recently, a connection between the circadian clock and autophagy has been reported in many systems [43–47]. For example, turnover of the clock protein brain and muscle ARNT-Like 1 (BMAL1) involves both proteasomal and autophagic activities [48]. As BMAL1 knock-down in human HF significantly prolongs anagen [18], it is conceivable that autophagy in the anagen hair matrix may impact on the peripheral clock in human HFs.

Because inhibiting autophagy by ATG5 silencing induces premature catagen and enhances hair MK apoptosis (Figs 4 and 5), autophagic flux in the anagen hair matrix appears to be important for anagen maintenance. That this is not only an ex vivo phenomenon but also clinically relevant is suggested by the fact that CQ can cause telogen effluvium in patients taking this antimalarial medication [22], which is caused by premature catagen induction [13,19]. Conversely, the principal ingredients (core mix) of a nutraceutical product used to treat hair loss potently promotes autophagy in organ-cultured human scalp HFs (Fig 6A–6E) and promotes anagen (Fig 7A and S3 Fig), but fails to do so in ATG5-silenced HFs (Fig 7B).

Taken together, our data support a scenario in which intrafollicular autophagy is eminently targetable for the therapeutic modulation of human hair growth. In line with this concept, another agent that positively regulates autophagy, caffeine [49,50], is sold as a hair growth–promoting cosmeceutical and has been shown to also prolong anagen and stimulate the proliferation of hair MK [51]. Autophagy inducers, which are the focus of intense ongoing research efforts [52–55], are therefore promising agents for the treatment of hair growth disorders and drug-induced hair loss phenomena characterized by premature catagen induction [56]. However, currently available chemical inducers of autophagy have limited specificity for the autophagic process and produce several autophagy-independent effects [55] that may also affect the HF cycle. Our genetically-modifiable HF model provides a suitable human test system for evaluating the efficacy and specificity of additional autophagy inducers in promoting hair growth.

Notably, HFs are continuously exposed to multiple, potentially noxious stimuli, ranging from contact with pathogens and the skin microbiome, UV light, and other DNA-damaging and/or oxidative stress-inducing external pressures, including drugs (many of which cause hair loss as an adverse effect) and internal stressors such as inflammation, metabolic disorders, and ageing [57–61]. To cope with this plethora of stressors, the human HF has established complex but highly efficient stress-response and stress-management systems [21,62–67]. The current data suggest that autophagy, a recognized stress-adaptive mechanism [2], is prominently enrolled into these intrafollicular stress-response/-management systems to such an extent that down-regulating autophagy does not permit human HFs to sustain their growth under conditions of stress (such as organ culture). This working concept can be followed up in vivo by studying human scalp skin xenotransplants onto immunocompromised mice, in which longer-term cycling of human HFs can be studied [16].

Interestingly, autophagy has also been found to be functionally important in regulating interfollicular epidermis (IFE) physiology and stress responses [68–71]. Thus, future functional comparative studies between intrafollicular and interfollicular autophagy may reveal an even more prominent function of autophagy in skin protection and function.

Our study also introduces and validates scalp HF organ culture as an instructive, clinically relevant preclinical tool for translational, autophagy-related studies in the human system ex vivo. This can now be used to evaluate the toxicity of candidate drugs with regard to how they affect autophagy in human (mini-)organs. For example, many drugs, including antidepressants, anticonvulsants, antihistamines, and anticancer agents, negatively impact on autophagy by reducing lysosomal function (lysosomotropy) [72]. High throughput assays developed for measuring this [72] can now be complemented in a second step by human HF organ culture as a much more clinically relevant drug toxicity–screening assay. Here, the efficacy and tissue toxicity of candidate autophagy-promoting and -inhibitory agents can also be assessed directly ex vivo.

Perhaps most importantly, the model introduced here provides the autophagy research community with an excellent tool for exploring the as yet insufficiently understood signals that regulate autophagy in human epithelial tissues as well as the functional roles of autophagy in a human (mini-)organ under both physiological and experimentally induced pathological conditions (e.g., chemotherapy [73], interferon-gamma treatment [64], ultraviolet radiation [66], and oxidative stress [63]).

## Materials and methods

### Ethics statement

Discarded human scalp HFs or skin samples were obtained with informed, written consent following the “Declaration of Helsinki Principles.” Full-length HFs used for the all the experiments were received and stored with ethical and institutional approval from the University of Manchester. A full list of patient numbers, sex, and age information is provided in S1 Table.

### Cell culture

Human osteosarcoma U2OS cells were acquired from the American Type Culture Collection (ATCC). Human NCTC 2544 keratinocytes were kindly provided by Dr. Barbara Marzani (Giuliani s.p.a, Milan, Italy). U2OS and NCTC 2544 were grown in Dulbecco’s Modified Eagle Medium High Glucose and Roswell Park Memorial Institute (RPMI) 1640 medium, respectively, containing 4.5 g/L D-Glucose, 4mM L-glutamine, 10% fetal bovine serum (FBS), and 0.25 mM sodium pyruvate. U2OS cells were adopted as an established excellent model cell system to evaluate pro-autophagic activity of natural products [36].

### IF microscopy

IF microscopy staining for localization and quantification of autophagy proteins LC3B, SQSTM1/p62, and ATG5 in situ was performed with anti-LC3B (D11) XP Rabbit mAb (Cell Signaling Technology), anti-SQSTM1/p62 (abcam), and anti-APG5L/ATG5 (EPR1755) (abcam) antibodies. Briefly, 5-μm-thick cryosections were fixed with cold acetone (−20 °C) for 10 min. After several washes in PBS, primary antibodies were incubated overnight at 4 °C. Alexa Fluor 555-conjugated donkey anti-Rabbit and Alexa Fluor 488-conjugated donkey anti-Mouse (Thermo Fisher) were adopted as secondary antibodies. Confocal images of HF derma papilla and of the area surrounding were taken by using a Confocal Microscope NIKON A1, the 20 × 0.25 and 60 × 1.40 numerical aperture objective lens. Quantitative immunohistomorphometry in defined reference area using standardized light exposure was performed with Image J (NIH) software, as described [29,64]. Microscopic hair cycle staging was performed as previously described, using Ki-67/TUNEL immunostaining and Masson Fontana histochemistry [15,17,29].

### Quantitative assessment of fluorescent signals in IF images

For the quantification of positive Ki-67– and TUNEL-positive cells, intramesenchymal TUNEL+ cells were excluded from our quantitative immunohistomorphometry analysis and only epithelial TUNEL-positive cells were counted. In fact, TUNEL-positive cells in the dermal papilla and connective tissue sheath is a well-recognized artifact of hair HF organ culture [15,29], because these regions of the human HF mesenchyme do not show apoptosis under physiological conditions [16].

Hair matrix cells were identified based on morphology and position relative to the dermal papilla [15,29].

Similarly, the quantifications of LC3B-, SQSTM1-, and ATG5-fluorescent signals were performed only on epithelial HF cells—specifically, the hair matrix cells and precortical matrix—while the connective tissue sheath and dermal papilla were excluded from analysis.

### TEM

Human scalp HFs were fixed in a mixture of 2% glutaraldehyde and 2% paraformaldehyde in 0.1 M cacodylate buffer for 2 h at room temperature and then processed for TEM as described in the literature [47]. Briefly, the samples have been washed in cacodylate buffer (0.1 M, pH 7.4), postfixed for 2 h with 1% osmium tetroxide in the same buffer, extensively washed again, and then incubated overnight in a 0.5% uranyl acetate aqueous solution in the dark. After washing, the sections have been dehydrated in a graded alcohol series, and after a final dehydration in 100% propylene oxide, they have been infiltrated with low viscosity Spurr resin overnight and polymerized for 48 h at 65 °C. Sections of about 70 nm were cut with a diamond knife (DIATOME) on a Leica EM UC6 ultramicrotome. Bright field TEM images have been collected with a Schottky field-emission gun FEI Tecnai G2 F20 (FEI, USA) transmission electron microscope operating at an acceleration voltage of 200 kV and equipped with a 2k × 2k Gatan Ultrascan (Gatan, USA) charge coupled device (CCD).

### Quantitative assessment of autophagic structures in TEM sections

For quantitative assessment of autophagy-related structures in TEM sections [74], we acquired images of both the cytoplasm and the autophagic structures at magnifications that allowed clear identification of compartments and delineation of profile boundaries. More than 20 fields were randomly recorded for each HF to quantify the number of AVs/field/follicle. Analysis and statistics were further performed on the average number of AVs per field from at least three independent HFs per condition.

#### ATG5 knock-down in organ-cultured human HFs

Micro-dissected human HFs were transfected with siRNA specific for *ATG5* or with a scrambled-oligo control acquired from Dharmacon (LQ-004374-00 and D-001810-01, respectively), as previously described [18].

#### Statistical analysis

One-way analysis of variance with Dunnett’s posttest and unpaired *t* test were performed using GraphPad-Prism Software (San Diego, CA). Excel spreadsheet in S1 Data contains numerical data and statistical analysis of Figs 2B, 2D, 3B, 3D, 3E, 4C, 4E, 5B, 5D, 5F, 5G, 6B, 6D, 6E, 6G, 7A, 7B and S2B, S2C, S3B and S3C Figs.

## Supporting information

S1 Fig Cultured human U2OS cells were treated 4 h with bafilomycin A1, a specific inhibitor of the vacuolar ATPase required for the fusion between autophagosomes and lysosomes in autophagy. Then, accumulation of lipidated LC3B protein (LC3-II) was assessed by two different antibodies against LC3B acquired from Cell Signaling Technology (CST) (cat. Number 3668) and MBL International (MBL) (cat. Number PM036). Although both CST and MBL antibodies recognized both lipidated (LC3-II) and non-lipidated (LC3-I) proteins, α-LC3B (D11) CST preferentially detected the LC3-II form. CST, Cell Signaling Technology; LC3, Light Chain 3; LC3B, Light Chain 3B; U2OS, human osteosarcoma epithelial U2OS cell line.
(TIF)

S2 Fig Cultured human NCTC 2544 keratinocytes were treated 6 h with vehicle or equimolar doses of spermidine and N^1^-methyspermidine (100 μM). (A) The levels of lipidated LC3 (LC3-II) and SQSTM1 were then assessed by immunoblotting analysis with specific antibody. Actin signals were adopted as a loading control. (B and C) Densitometry analysis of protein signals is reported as relative protein levels normalized by ACTIN. Vehicle sample value was set to 1. Shown as mean ± SEM, *n* = 3. *P < 0.05 and **P < 0.01, compounds versus vehicle. The underlying numerical data are provided in S1 Data. ACTIN, actin beta; LC3, Light Chain 3; NCTC, human keratinocyte NCTC 2544 cell line; SQSTM1, sequestosome 1.
(TIF)

S3 Fig (A) Representative image showing Ki-67/TUNEL immunostaining and Masson Fontana histochemistry in HFs treated with the principal ingredients (core mix) of a commercial anti–hair loss product or vehicle. (B and C) The number of proliferative and apoptotic MKs below the widest part of the dermal papilla (Auber’s line) in vehicle- and core mix–treated HFs from three diverse donors were calculated as the relative percentage of Ki-67– and TUNEL-positive cells in core mix–treated HFs compared with vehicle-treated HFs. Shown as fold difference versus vehicle ± SEM. *P < 0.05 and ***P < 0.001, core mix versus Control. As TUNEL-positive cells in the dermal papilla and connective tissue sheath is a well-recognized artifact of HF organ culture [15,16,29], intramesenchymal TUNEL-positive cells were excluded from the quantitative analysis. The underlying numerical data are provided in S1 Data. HF, hair follicle; MK, matrix keratinocyte; TUNEL, Terminal deoxynucleotidyl transferase dUTP Nick End Labeling.
(TIF)

S1 Table Patient number, sex, and age information of human donors adopted in this study. (DOCX)

S1 Data Excel spreadsheet containing, in separate sheets, the underlying numerical data and statistical analysis for figure panels Figs 2B, 2D, 3B, 3D, 3E, 4C, 4E, 5B, 5D, 5F, 5G, 6B, 6D, 6E, 6G, 7A, 7B and S2B, S2C, S3B and S3C Figs. (XLSX)

## Abbreviations

ATCC: American Type Culture Collection
*ATG5*: autophagy-related gene 5
ATG8: autophagy-related protein 8
AVd: late/degradative autophagic vacuole
AVi: initial autophagic vacuole
BMAL1: brain and muscle ARNT-Like 1
CCD: charge coupled device
CQ: chloroquine
FBS: fetal bovine serum
FGF: fibroblast growth factor
HF: hair follicle
IF: immunofluorescence
IFE: interfollicular epidermis
iRNA: interference RNA
KGF: keratinocyte growth factor
LC3: Light Chain 3
LC3B: Light Chain 3B
MK: matrix keratinocyte
RPMI: Roswell Park Memorial Institute
siRNA: small interfering RNA
SQSTM1/p62: sequestosome 1
TEM: transmission electron microscopy
TUNEL: Terminal deoxynucleotidyl transferase dUTP Nick End Labeling
