# Supplementary figures and images for: Autophagy is essential for maintaining the growth of a human (mini-)organ: Evidence from scalp hair follicle organ culture
Source: PLoS Biol. 2018 Mar 28;16(3):e2002864. doi: 10.1371/journal.pbio.2002864 (PMC5891029; doi:10.1371/journal.pbio.2002864)

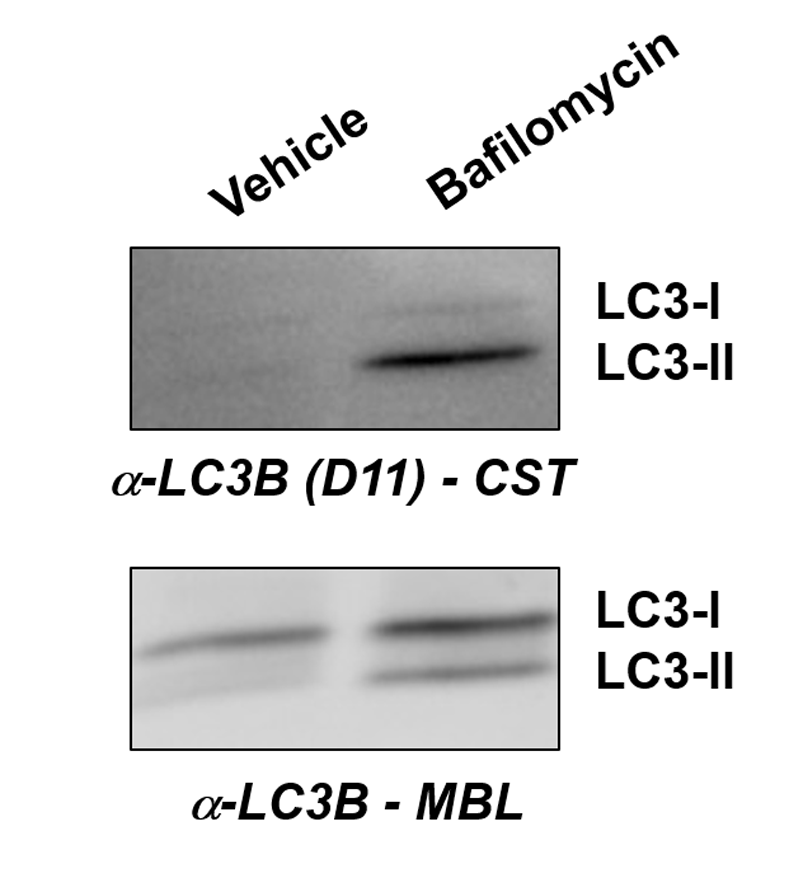

Supplement: S1 Fig — Then, accumulation of lipidated LC3B protein (LC3-II) was assessed by two different antibodies against LC3B acquired from Cell Signaling Technology (CST) (cat. Number 3668) and MBL International (MBL) (cat. Number PM036). Although both CST and MBL antibodies recognized both lipidated (LC3-II) and non-lipidated (LC3-I) proteins, α-LC3B (D11) CST preferentially detected the LC3-II form. CST, Cell Signaling Technology; LC3, Light Chain 3; LC3B, Light Chain 3B; U2OS, human osteosarcoma epithelial U2OS cell line. (TIF) [file pbio.2002864.s001.tif]

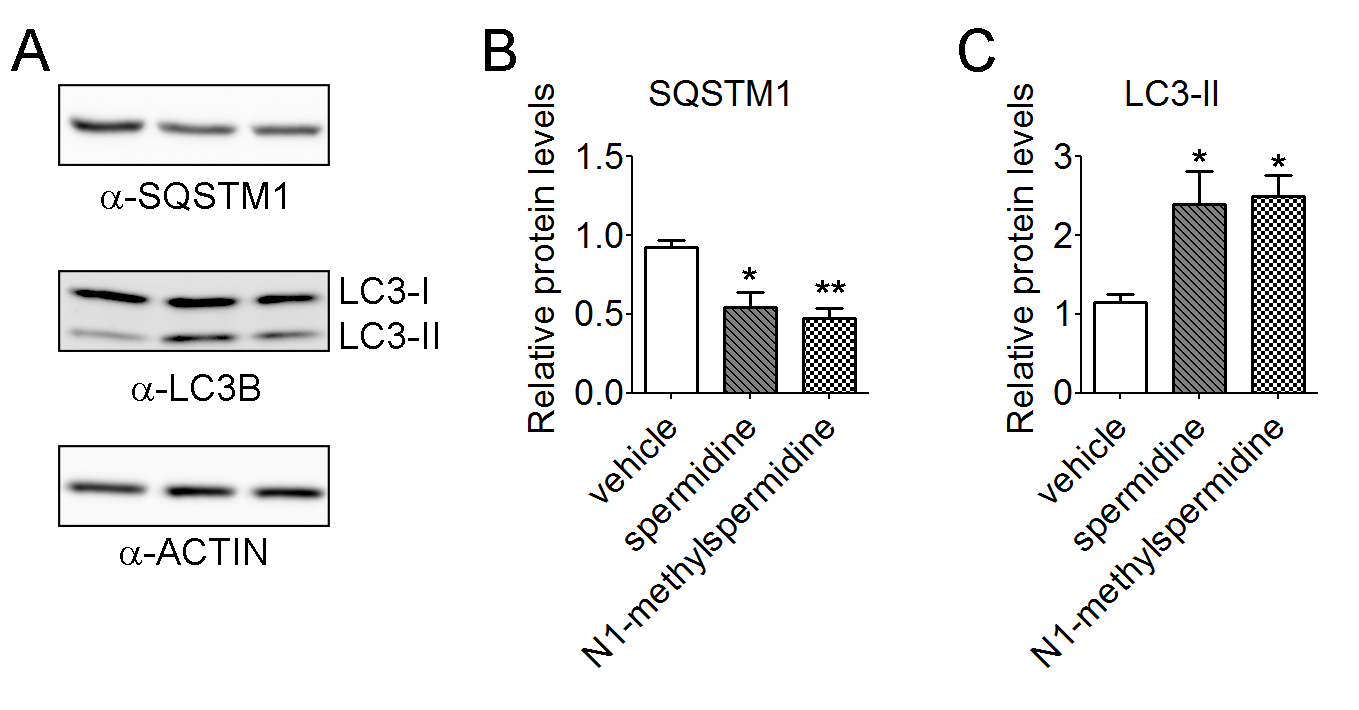

Supplement: S2 Fig — (A) The levels of lipidated LC3 (LC3-II) and SQSTM1 were then assessed by immunoblotting analysis with specific antibody. Actin signals were adopted as a loading control. (B and C) Densitometry analysis of protein signals is reported as relative protein levels normalized by ACTIN. Vehicle sample value was set to 1. Shown as mean ± SEM, n = 3. *P < 0.05 and **P < 0.01, compounds versus vehicle. The underlying numerical data are provided in S1 Data. ACTIN, actin beta; LC3, Light Chain 3; NCTC, human keratinocyte NCTC 2544 cell line; SQSTM1, sequestosome 1. (TIF) [file pbio.2002864.s002.tif]

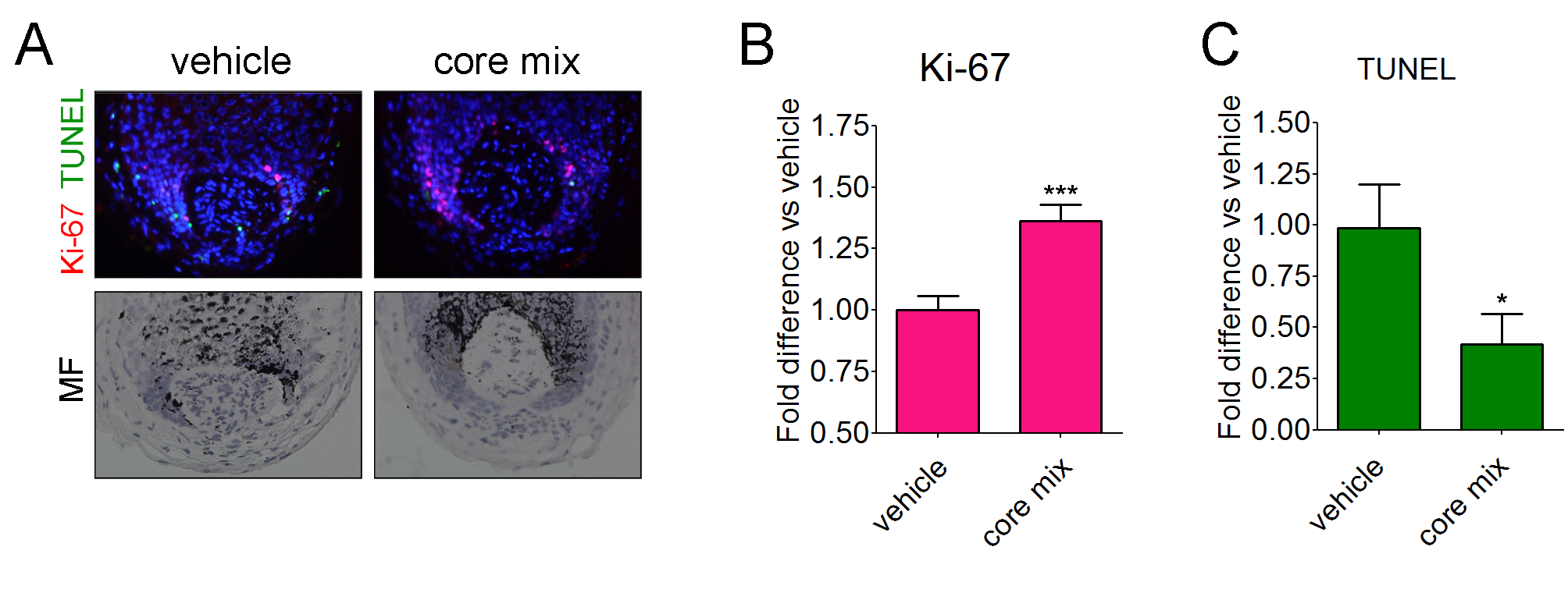

Supplement: S3 Fig — (A) Representative image showing Ki-67/TUNEL immunostaining and Masson Fontana histochemistry in HFs treated with the principal ingredients (core mix) of a commercial anti–hair loss product or vehicle. (B and C) The number of proliferative and apoptotic MKs below the widest part of the dermal papilla (Auber’s line) in vehicle- and core mix–treated HFs from three diverse donors were calculated as the relative percentage of Ki-67– and TUNEL-positive cells in core mix–treated HFs compared with vehicle-treated HFs. Shown as fold difference versus vehicle ± SEM. *P < 0.05 and ***P < 0.001, core mix versus Control. As TUNEL-positive cells in the dermal papilla and connective tissue sheath is a well-recognized artifact of HF organ culture [15,16,29], intramesenchymal TUNEL-positive cells were excluded from the quantitative analysis. The underlying numerical data are provided in S1 Data. HF, hair follicle; MK, matrix keratinocyte; TUNEL, Terminal deoxynucleotidyl transferase dUTP Nick End Labeling. (TIF) [file pbio.2002864.s003.tif]
