# Supplementary material for: Autophagy is essential for maintaining the growth of a human (mini-)organ: Evidence from scalp hair follicle organ culture
Source: PLoS Biol. 2018 Mar 28;16(3):e2002864. doi: 10.1371/journal.pbio.2002864 (PMC5891029; doi:10.1371/journal.pbio.2002864)
Supplement: S1 Table — (DOCX) [file pbio.2002864.s004.docx]

**S1 Table**.

Patient number, sex and age information of human donors adopted in this study.

| **Patient #** | **Age** | **Sex** | **Experiment** |
| --- | --- | --- | --- |
| 1 | 68 | Male | Anagen/Catagen comparison |
| 2 | 49 | Male | Anagen/Catagen comparison |
| 3 | 34 | Male | Anagen/Catagen comparison |
| 4 | 53 | Male | ATG5 silencing experiments |
| 5 | 42 | Male | ATG5 silencing experiments |
| 6 | 29 | Male | ATG5 silencing experiments |
| 7 | 30 | Male | ATG5 silencing experiments |
| 8 | 71 | Male | Chloroquine treatment ( h, 10µM) |
| 9 | 36 | Male | Chloroquine treatment ( h, 10µM) |
| 10 | 38 | Male | Chloroquine treatment ( h, 10µM) |
| 11 | 29 | Male | ATG5 silencing experiments in presence or absence of autophagy inducer mix (7.9ng/mL *Galeopsis segetum* extract, 0,5µM Met-spermidine, 19,91 ng/mL Biotin) |
| 12 | 24 | Male | ATG5 silencing experiments in presence or absence of autophagy inducer mix (7.9ng/mL *Galeopsis segetum* extract, 0,5µM Met-spermidine, 19,91 ng/mL Biotin) |
| 13 | 27 | Male | ATG5 silencing experiments in presence or absence of autophagy inducer mix (7.9ng/mL *Galeopsis segetum* extract, 0,5µM Met-spermidine, 19,91 ng/mL Biotin) |
| 14 | 58 | Female | Hair follicle stage evaluation in presence of autophagy inducer mix (7.9ng/mL   *Galeopsis segetum* extract, 0,5µM Met-spermidine, 19,91 ng/mL Biotin). |
| 15 | 61 | Female | Hair follicle stage evaluation in presence of autophagy inducer mix (7.9ng/mL   *Galeopsis segetum* extract, 0,5µM Met-spermidine, 19,91 ng/mL Biotin). |
| 16 | 50 | Male | Hair follicle stage evaluation in presence of autophagy inducer mix (7.9ng/mL   *Galeopsis segetum* extract, 0,5µM Met-spermidine, 19,91 ng/mL Biotin). |
